# Vapor deposition routes to conformal polymer thin films

**DOI:** 10.3762/bjnano.8.76

**Published:** 2017-03-28

**Authors:** Priya Moni, Ahmed Al-Obeidi, Karen K Gleason

**Affiliations:** 1 Department of Materials Science and Engineering, Massachusetts Institute of Technology, 77 Massachusetts Ave, Cambridge, MA 02139, USA; 2 Department of Chemical Engineering, Massachusetts Institute of Technology, 77 Massachusetts Ave, Cambridge, MA 02139, USA

**Keywords:** conformal, polymers, thin films, vapor deposition

## Abstract

Vapor phase syntheses, including parylene chemical vapor deposition (CVD) and initiated CVD, enable the deposition of conformal polymer thin films to benefit a diverse array of applications. This short review for nanotechnologists, including those new to vapor deposition methods, covers the basic theory in designing a conformal polymer film vapor deposition, sample preparation and imaging techniques to assess film conformality, and several applications that have benefited from vapor deposited, conformal polymer thin films.

## Review

### Introduction

Conformal coverage is achieved when a film of uniform thickness precisely follows the geometry of the underlying substrate. Conformal coatings allow for surface properties to be optimized independently from the choice of the bulk material and shape of the substrate. Conformality has become an increasingly important characteristic in the fabrication of optoelectronic and medical devices having high aspect ratio features, 3D geometries, and textured/nanostructured surfaces. Conformal coating methods are also desired for modifying the internal surfaces of porous materials, including membranes, foams, and textiles, or irregular surface geometries, as well as for encapsulating fibers, nanowires, or particles [1]. For example, tailoring the surface energy of the pore walls of a separation membrane without obstructing the pore can enhance the passage of the desired liquid or gas [2–4]. Conformal coatings can also ensure that micro or nano-device properties (e.g., conductance, capacitance) do not vary due to large thickness variations [5–7].

**Figure 1:**
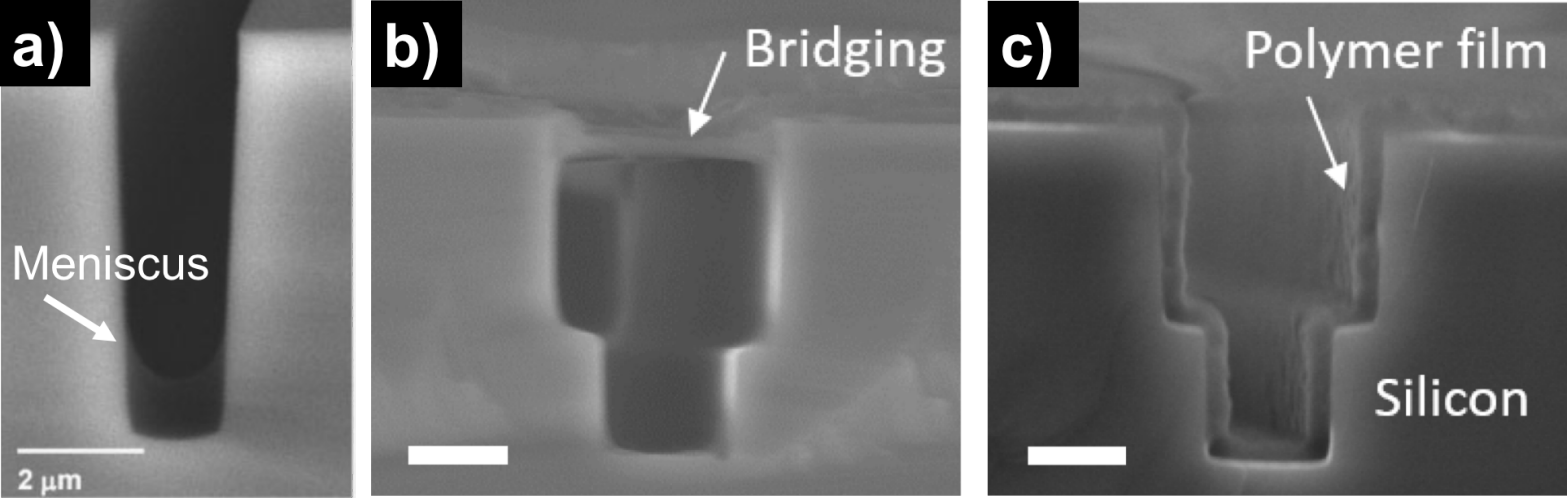
Micro-trenches with polymer coatings by a) solution with low substrat–interface energy, b) solution with high substrate–interface energy and c) iCVD (scale bar 2 µm). a) Reproduced with permission from [8], Copyright 2008 John Wiley and Sons. b),c) Reproduced with permission from [9], Copyright 2016 Massachusetts Institute of Technology.

One motivation for vapor phase synthesis of polymer thin films over traditional solution methods (e.g., spin casting, dip coating) is the ability to form conformal films on high aspect ratio structures, as seen in Figure 1. In traditional methods, polymers are pre-synthesized and dispersed in a solvent. This solution is then spread on the substrate of interest, typically by dip or spinning coating, and dried so that only the polymer, in film form, remains on the surface. While this technique works reasonably well for flat substrates, the interaction energies between solution components coupled with its overall interfacial energy with the substrate can result either meniscus formation inside a feature (Figure 1a) or capillary bridge formation over a feature (Figure 1b). By contrast, vapor phase techniques are controlled by the individual adsorption of small molecules and their subsequent surface reaction to form a polymer film. In this case, the only interaction energy of concern is between the molecule and an available surface. Controlling the reactor conditions to ensure the Knudsen number is greater than unity (i.e., the mean free path of the molecule is greater than the relevant substrate geometric length scale) results in molecular adsorption deep in a structure so that the final film evenly coats the substrate geometry (Figure 1c) [1].

Several chemical vapor deposition (CVD) techniques result in highly conformal polymer films. For instance, emerging techniques such as molecular layer deposition (MLD) and oxidative CVD (oCVD) form conformal metalucone and step-growth polymer films [10–11]. However, no systematic studies of conformality have been devoted solely to these techniques thus far. Practitioners of MLD can look at existing models for its inorganic analogue, atomic layer deposition (ALD), as a starting point for studying conformal MLD films [12]. This review will focus on two, well-studied, conformal polymer CVD techniques: parylene CVD and initiated CVD (iCVD), with both deriving from free radical polymerization mechanisms. The four parts of this review will address reaction mechanisms of the aforementioned techniques, necessary deposition conditions for conformal film growth, imaging conformal polymer films, and finally applications for conformal polymer films.

### Reaction mechanisms

#### Parylene CVD

**Figure 2:**
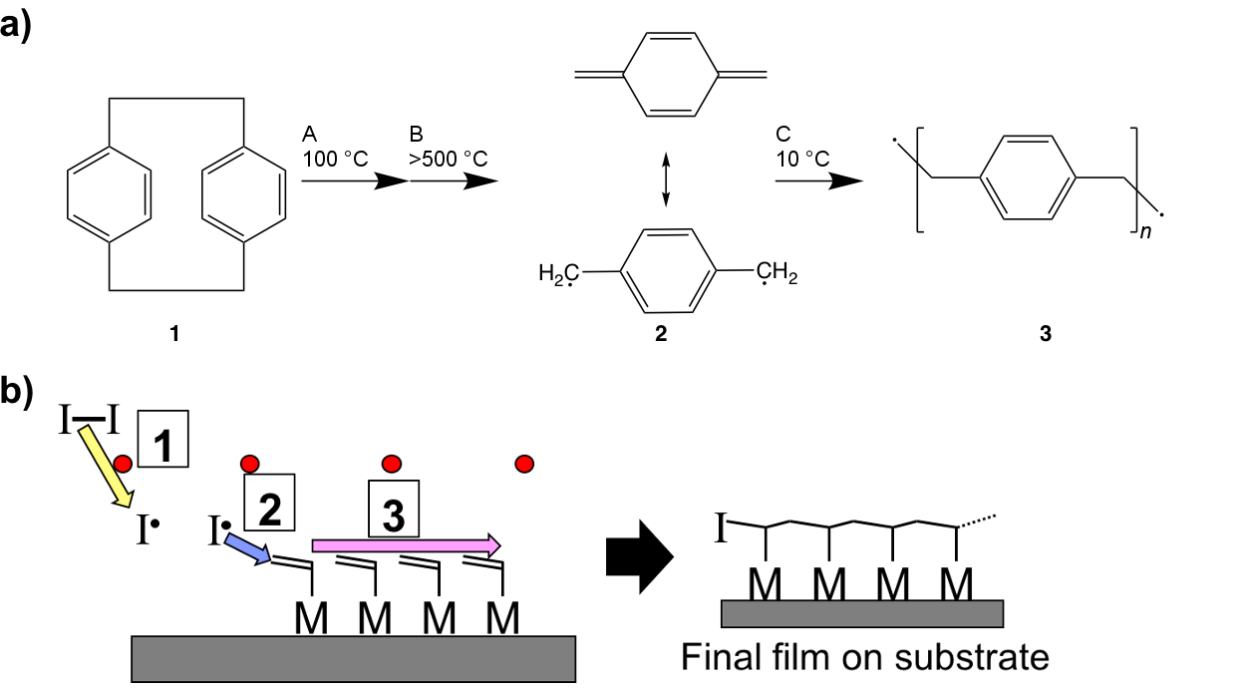
a) Mechanism of parylene CVD. **(1)** [2,2]paracyclophane **(2)** *p*-xylylene diradical intermediate **(3)** poly[*p*-xylene]; A) Sublimation, B) pyrolysis, C) substrate adsorption. b) Mechanism of iCVD: 1) decomposition of initiator by hot filament, 2) initiator attack of adsorbed monomer, 3) propagation to form polymer film.

Parlyene CVD is a well-established, free radical polymerization technique that results in poly[*p*-xylene] films [13]. The reaction mechanism proceeds as shown in Figure 2a, where [2,2]paracyclophane (22PCP) molecules are first sublimed, then thermally cracked at >500 °C to form two, resonance stabilized *p*-xylylene diradicals that eventually adsorb on a substrate near room temperature and react to form poly[*p*-xylene] [13–14]. Functionalized derivatives of the 22PCP monomer precursor enable the introduction of new chemistries into the final poly[*p*-xylene] structure such as halogens, amines, and esters [15–16].

#### Initiated CVD

iCVD is another free radical polymerization technique where instead of a single reactive species, a monomer and an initiating radical are needed to form the final polymer film. As seen in Figure 2b, gas phase monomers containing a reactive bond first adsorb on the substrate near room temperature. An initiator, such as di-*tert*-butyl peroxide is thermally cleaved by a hot filament (≈250 °C) and the resulting radicals collide with surface adsorbed monomers to initiate polymerization. The most common monomers polymerizable by iCVD are acrylates, methacrylates, and other vinyl (>C=C<) containing monomers [17–18]. However, acetylenic (–C≡C–) monomers have been polymerized as well [19].

### The effect of deposition conditions

Depending on the conditions used, a CVD process can vary from extremely conformal to extremely non-conformal (planarization). Therefore, it is important to know what factors enable conformal film deposition and how these are related to the deposition conditions used.

Thin film depositions on well-defined micron-sized trench structures are often used to study the process’ conformality. When studying the conformality on a trench structure, step coverage (SC) and side wall coverage (SWC) are the most important properties to assess. SC and SWC are defined in Equation 1 and Equation 2, respectively:

[1]
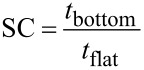


[2]
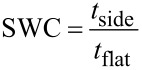


where *t*_bottom_, *t*_side_, and *t*_flat_ are the film thicknesses at the bottom of trench, side wall of a trench, and flat surface at the top of a trench, respectively. For perfect conformality, SC and SWC should be unity, where any deviation indicates some degree of non conformality. Several theoretical models regarding conformal depositions of parylene-CVD and iCVD in high aspect ratio structures have been published elsewhere [1,3,20–21]. However all systems share a common dependence on the sticking probability, Γ, or the probability that a gas molecule will chemisorb on a surface [22]. In CVD reactions, film conformality improves as reactant sticking probabilities decreases since this enables gas diffusion deeper into deep structures [1,21]. Γ has many dependencies, but the fractional coverage of chemisorbed species, θ, and various chemical reaction rates, *R*, can play a significant role for polymer growth systems [3]. In general, increasing θ and/or reducing *R* results in a reduced Γ. In order to develop process optimization strategies for deposition process having more than one gas phase reactant, it is important to determine which species has the Γ which controls the degree of conformality.

#### Parlyene CVD

During parylene CVD, the adsorption of a single *p*-xylylene diradical usually results in no chemical reaction. However, when a *p*-xylylene diradical collides with a cluster of two adsorbed diradicals, it can react to form a new, heavy chain that does not desorb from the surface [13]. Analysis by Fortin and Lu using the chemisorption model and Langmuir isotherm determined the following relationship between Γ and θ

**Figure 3:**
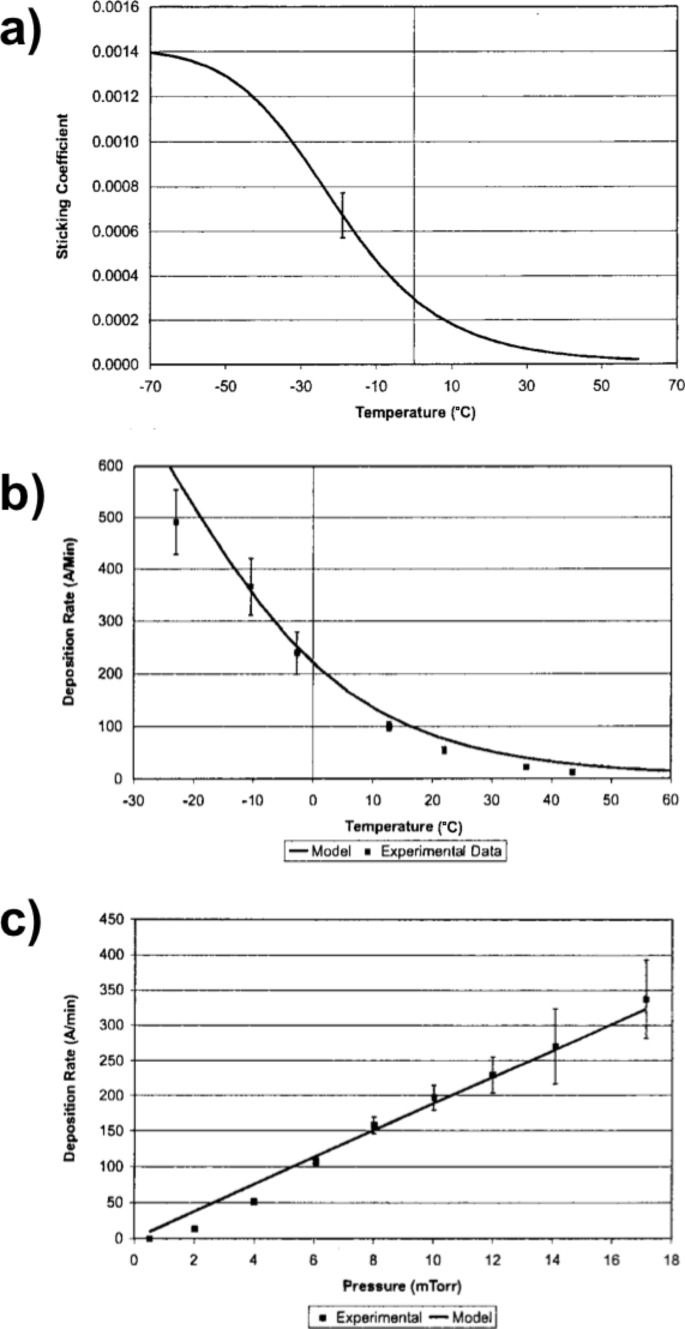
a) Sticking coefficient of *p*-xylylene diradicals as a function of temperature, b) deposition rate as function of temperature at pressure = 4.0 mTorr, c) deposition rate as function of pressure at temperature = 22 °C. Adapted with permission from [20], copyright 2002 American Chemical Society.

[3]
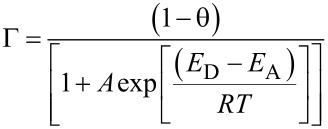


where *E*_D_ and *E*_A_ are the energies of desorption and adsorption, respectively, *R* is the gas constant, *T* is temperature, and *A* is a pre-exponential factor [20]. In parylene depositions, gaseous diradical monomers can chemisorb directly on the reactive chain ends, thus growing the chain while still maintaining the active chain ends. This results in (1 − θ), the fraction of available sites for chemisorption, remaining essentially constant during the deposition, since it is proportional to the number of growing chains [13]. To reduce Γ, the overall number of sites for chemisorption must decrease, meaning that the density of monomers adsorbed on the substrate surface must be reduced. This can be achieved by reducing the partial pressure of the monomer either by introducing an inert gas flow or by reducing the total pressure of the deposition. Another approach to reduce the sticking coefficient is to increase the substrate temperature to hinder monomer adsorption. The functional dependence of temperature on Γ is seen in Equation 3 and plotted in Figure 3a. While a reduction both in chamber pressure or increase in substrate temperature allow for more conformal film growth, Figure 3b and Figure 3c show that the deposition rate also decreases in these conditions. Thus highly conformal processes come at the expense of fast film growth rates.

#### Initiated CVD

**Figure 4:**
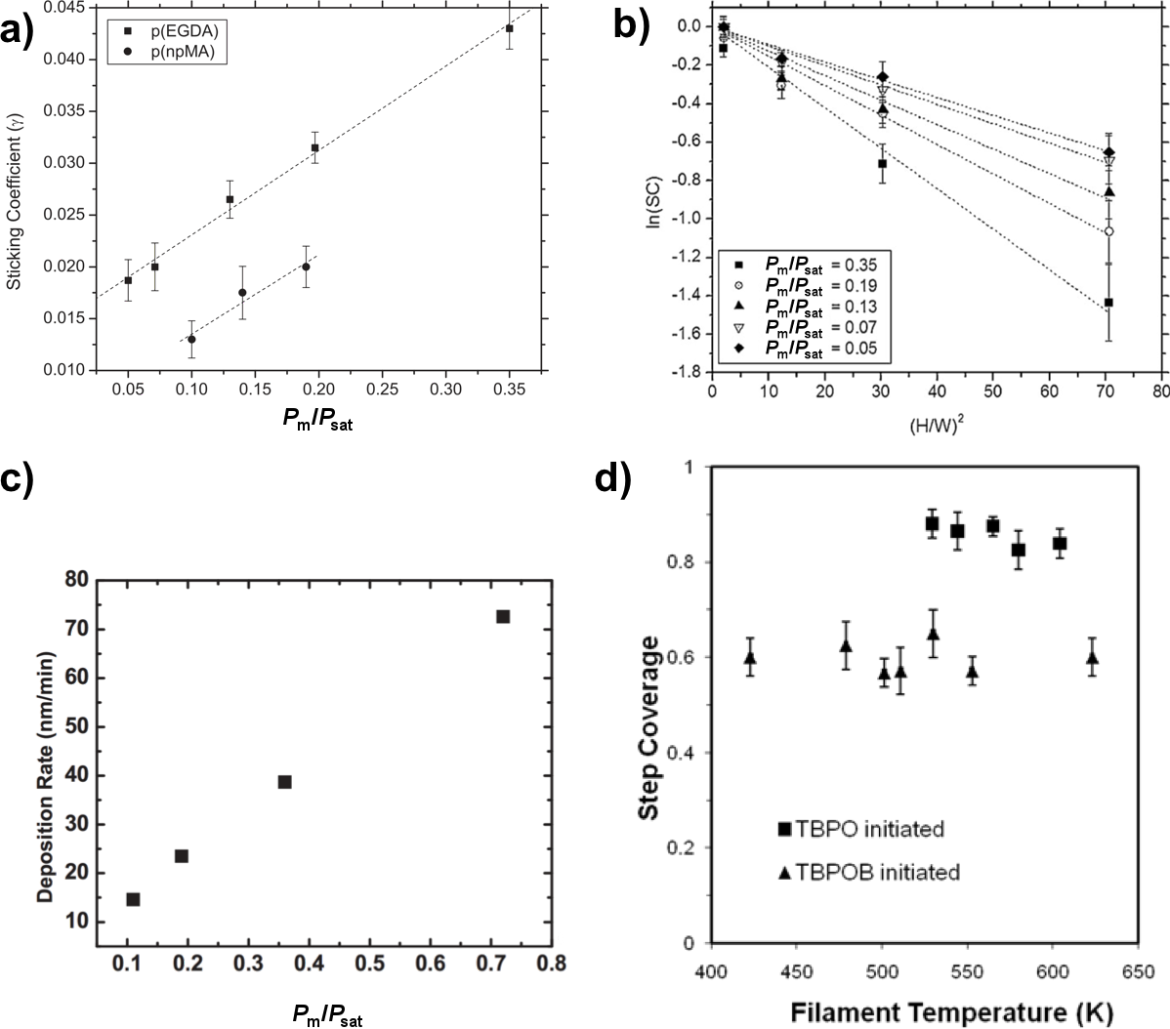
a) Sticking coefficients of *tert*-butoxy initiator radicals as function of *P*_m_/*P*_sat_ and monomer type where EGDA, a di-acrylate monomer, has a higher Γ than npMA, a methacrylate monomer, due to the presence of a second reactive moiety. b) Step coverage as a function of trench aspect ratio at varying *P*_m_/*P*_sat_ values. c) iCVD deposition rate as function of *P*_m_/*P*_sat_. d) Step coverage as a function of filament temperature and type of initiator. Parts a) and b) reprinted with permission from [25], copyright 2010 John Wiley and Sons. c) Reprinted with permission from [21], copyright 2008 John Wiley and Sons. d) Reprinted with permission from [24], copyright 2011 American Chemical Society.

During iCVD, clusters of unreacted monomers adsorb on the substrate and quickly polymerize upon the impingement of an initiator radical. The initiator radicals are quite volatile and are expected to have negligible adsorption on the bare surface. Additionally, once a monomer undergoes polymerization, it is no longer a site for initiator chemisorption. Therefore, the number surface sites available for the initiator is directly related to the monomer fractional surface coverage given by *P*_m_/*P*_sat_ where *P*_m_ is the partial pressure of the monomer in the chamber and *P*_sat_ is the monomer’s saturation pressure under the given deposition conditions [23]. Operating at lower *P*_m_/*P*_sat_ values thus reduces the sticking probability of the initiator radical, as seen in Figure 4a. In Figure 4b, a general trend of decreasing step coverage is seen with an increase in aspect ratio. However, by decreasing the *P*_m_/*P*_sat_, step coverages closer to unity are possible even at higher aspect ratios. Finally, like parylene CVD, conformal deposition conditions in iCVD come at the cost of deposition rate. In Figure 4c, a positive relationship between *P*_m_/*P*_sat_ and deposition rate is demonstrated. In iCVD, conformality can be maintained for rates up to ≈50 nm/min [21].

To confirm that conformality is controlled by the sticking probability of the initiator, the same monomer, cyclohexylmethacrylate, was iCVD polymerized holding *P*_m_/*P*_sat_ fixed using two different initiators [24]. The first initiator was *tert*-butyl peroxide (TBPO) which decomposes over the heated filament to give two *tert*-butoxy radicals. The second initiator was *tert*-butyl peroxybenzoate (TBPOB) which decomposes to give one *tert*-butoxy radical and one high molecular weight benzoate radical. Figure 4d shows that the sticking coefficients for the higher molecular weight radicals produced for TBPOB, are consistently greater than for TBPO. The sticking coefficient of both initiator radicals is independent of filament temperature. Since the filament temperature determines the fraction of initiators cleaved to radicals, the sticking probability of the initiator radicals is independent of their gas phase concentration. This study confirms that volatile initiators are desirable for conformal iCVD growth.

**Diffusion and reaction controls.** As aspect ratios of geometries increase, both the rate of reaction and diffusion of reactants down the feature play a much larger role in the process conformality. Uniform film growth requires reactants to be readily available at all point down a high aspect ratio structure. However, if the rate of propagation for chain growth is much higher than the rate monomer diffusion, this can result in a concentration profile down the geometry. In this case, the monomer sticking probability must also be considered.

Asatekin et al. studied the impact of the Thiele modulus, Φ, which compares the consumption of a reactant to its replenishment by diffusion, on conformality of iCVD film formation [3]. For a pore of depth *L* and radius *r*, the following equation was derived for the iCVD system

[4]
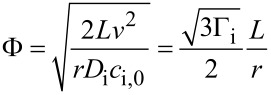


Where *v* is the deposition rate, and *D*_i_, *c*_i,0_, and Γ_i_ are the diffusivity, concentration at pore entrance, and sticking coefficient of species i respectively. The Thiele modulus can then be used to modify Fick’s second law to yield the following equation describing the concentration profile at position *x* down the pore’s length:

[5]
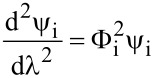


with dimensionless variables λ = *x*/*L* and ψ_i_ = *c*_i_/*c*_i,0_, where *c*_i_ is the concentration of species i at position *x* [1]. The combined impact of the monomer concentration profile, ψ_M_, and initiator concentration profile, ψ_I_, on the step coverage at the bottom of the pore is then given by Equation 6 [1].

[6]
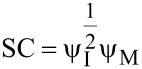


**Figure 5:**
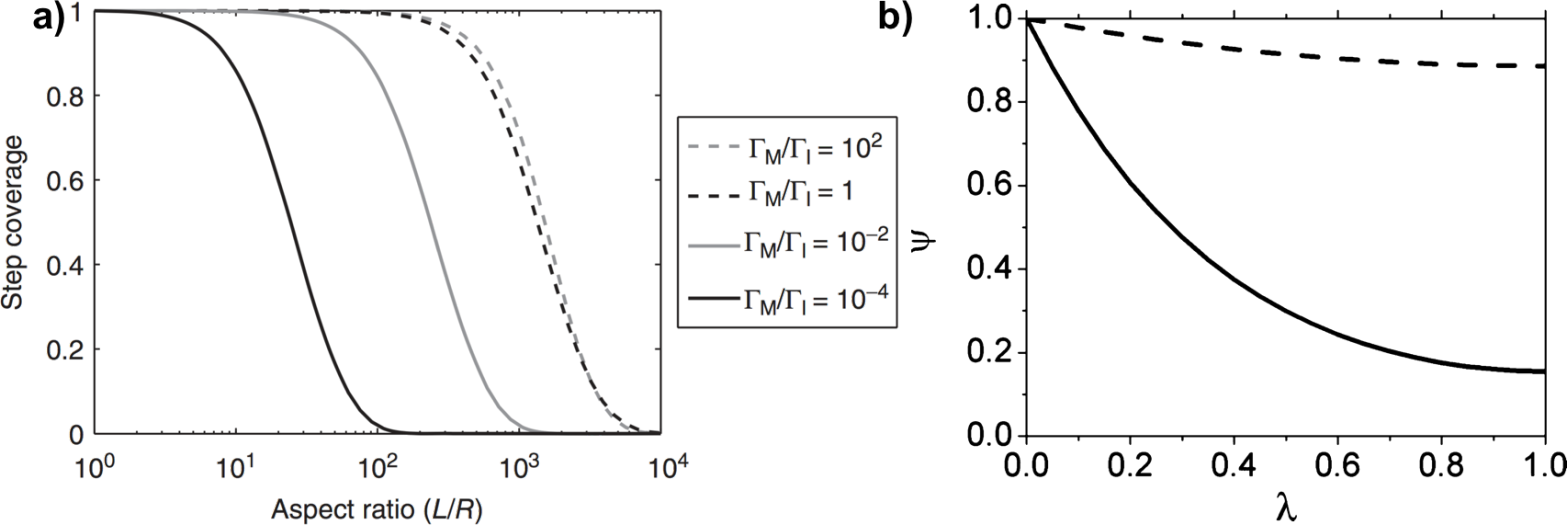
a) Step coverage as function of aspect ratio and ratio of sticking coefficients from numerical solutions of Equation 6. b) Concentration profile of perfluorodecyl acrylate (solid line) and divinyl benzene (dashed line) down a pore. a) Reprinted with permission from [1], copyright 2015 John Wiley and Sons. b) Reprinted with permission from [3], copyright 2011 American Chemical Society.

The ratio of monomer to initiator sticking coefficients has a substantial impact on the final value of step coverage for a given aspect ratio. Numerical solutions to Equation 6 are plotted in Figure 5a. High step coverage at higher aspect ratios requires the monomer sticking coefficient to be substantially smaller than the initiator sticking coefficient.

The propagation rate also affects the monomer sticking probability and concentration profile within a pore. If the Φ_M_ < 1, the monomers in a pore are continuously replenished meaning the monomer concentration profile within the pore is essentially constant [1,3]. This can occur in reactions with low radical reaction propagation rates, like the polymerization of vinyl monomers, which results in very low monomer sticking coefficients. An example of this is seen in Figure 5b, where the dashed line representing the divinyl benzene concentration profile is relatively constant. Thus only the initiator sticking probability affects step coverage, as given by the following relationship [1,21].

[7]
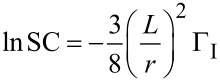


However, if Φ_M_ >1, a gradient in the monomer concentration develops down the length of the pore. In this regime, polymerization consumes monomer faster than monomers can be replenished by diffusion down the pore. This is common for methacrylate and acrylate monomers, which have high radical polymerization rates [3]. An example of the increased reduction of monomer concentration down a pore is seen for perfluorodecyl acrylate, the solid in line Figure 5b. The high propagation rate also results in a corresponding increase in the monomer sticking coefficient, since monomer chemisorption on a growing surface chain has become kinetically favored. Step coverage is poor in this case, as bottlenecks often form, completely obstructing the pores.

### Assessing conformality

**Figure 6:**
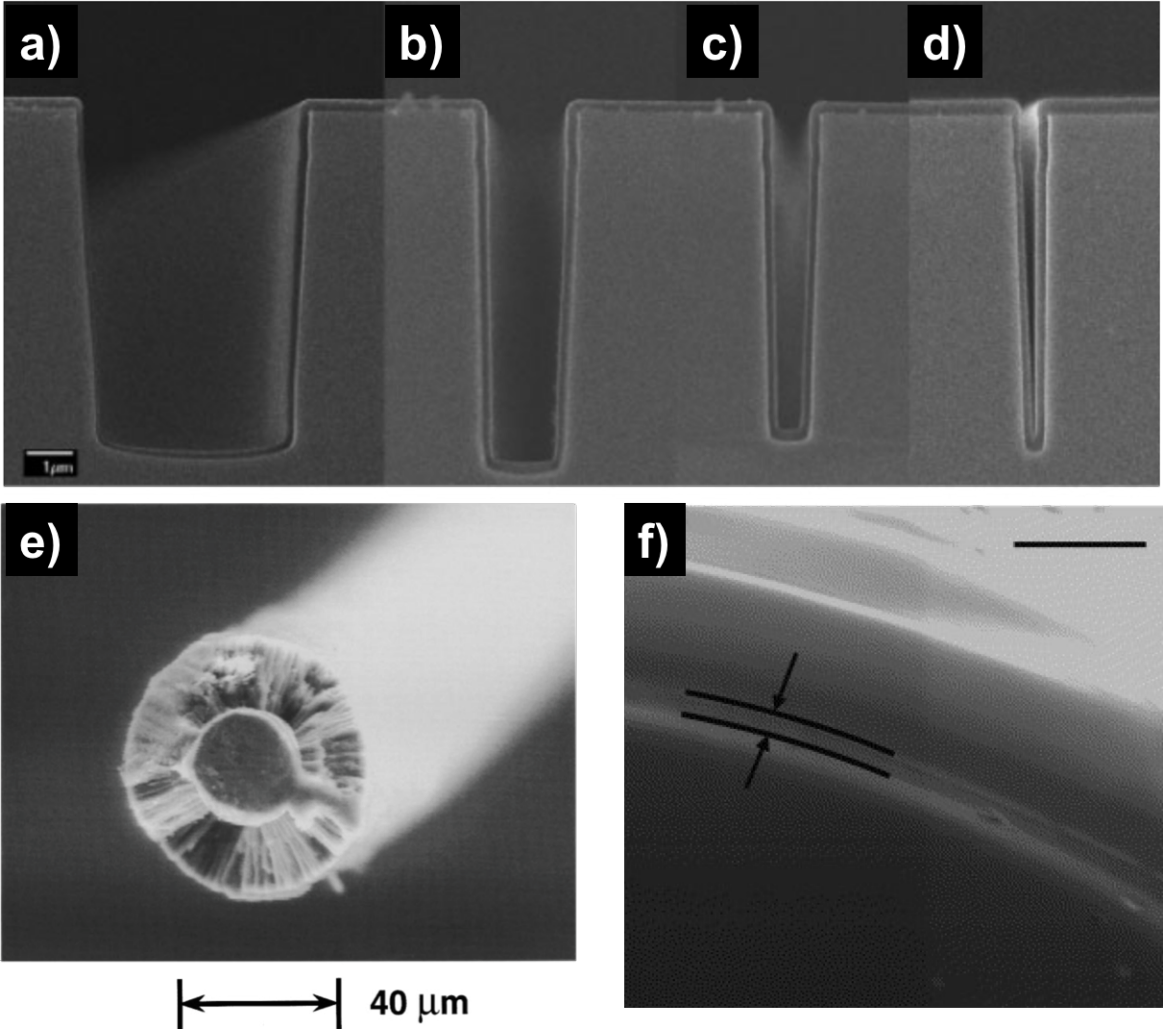
SEM images of iCVD pEGDA on micro-trenches with aspect ratios of a) 1.4 b) 3.5, c) 5.5 and d) 8.4. e) Cross-sectional SEM of 25 µm stainless-steel wire with 16 µm fluoropolymer coating formed via iCVD. f) iCVD pDMAMS on nylon fiber ion beam ablated to reveal substrate, scale bar is 1 µm. Parts a), b), c) and d) reprinted with permission from [25], copyright 2010 Wiley. e) Reprinted with permission from [26], copyright 1996, AIP Publishing LLC. f) Reprinted with permission from [27], copyright 2007, Elsevier.

Experimentally, conformality is determined using electron microscopy. Depending on the substrate structure, material, polymer film thickness, and final application, additional or varied techniques may be needed. The simplest case is a polymer film thicker than 200 nm on a micron-sized structure with sufficient material atomic number contrast (e.g., Si). In this case, physically cleaving the sample and taking a cross-sectional scanning electron microscopy (SEM) image will show how the film is coated on the substrate, as seen in Figure 1c and Figure 6a–d. Conformality of a given process can be assessed by creating a series of trenches of varying aspect ratios, as seen in the top panel of Figure 6. However, conformal depositions are desirable on more geometries than just trenches. For instance, an insulating polymer film uniformly enveloping a conductive wire may be required for an application. As seen in Figure 6e, a simple cross section of the wire can reveal the conformal coating [26]. Imaging a series of cross sections can inform conformality along the length of the wire. Not all complex substrates are amenable to forming physical cross sections. In this case, ion or electron beam ablation can expose the substrate so that the film–substrate interface can be imaged [27–28]. In Figure 6f, an iCVD coated textile fiber has been ion beam ablated to reveal the conformal polymer film [27].

**Figure 7:**
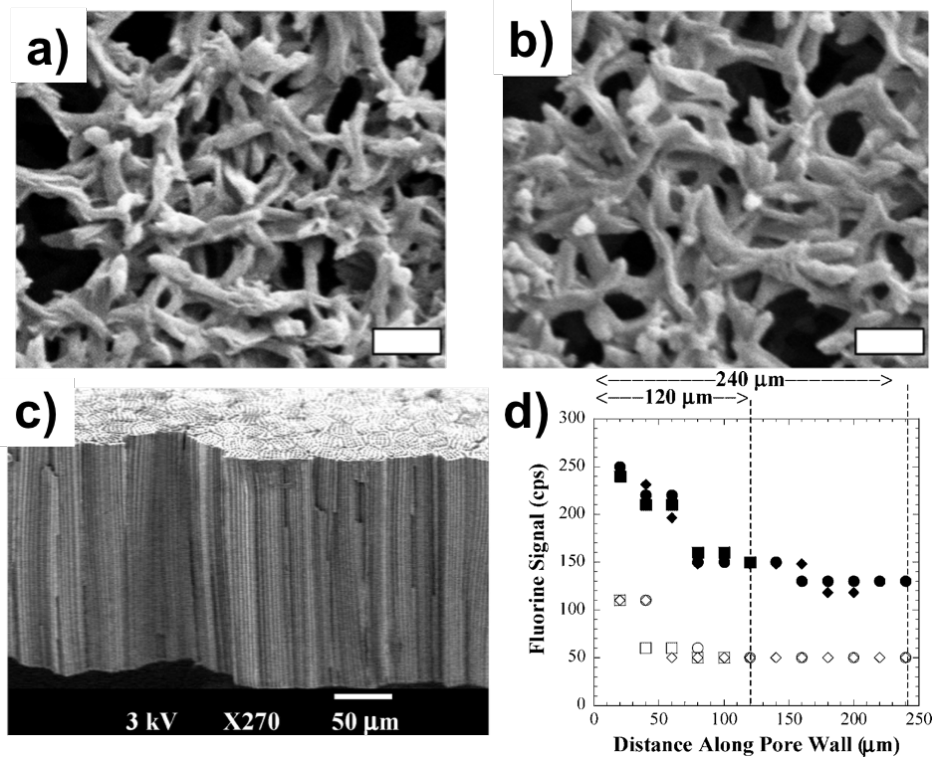
SEM of Nylon membranes a) uncoated and b) coated with 10 nm of iCVD pDVB (scale bar 1 µm). c) Cross-sectional SEM of capillary pore membrane. d) EMPA fluorine signal down 3 µm diameter pore with 2 minute deposition (unfilled) and 5 minute deposition (filled). Parts a) and b) reprinted with permission from [2], copyright 2016 Elsevier. Parts c) and d) reprinted with permission from [4], copyright 2008 American Chemical Society.

As substrates become more complex and polymer film thicknesses fall below 100 nm, verifying film conformality becomes increasingly difficult. Insufficient Z contrast and charging effects makes SEM cross sections difficult to analyze. Using a focus ion beam (FIB) system to make transmission electron microscopy (TEM) samples is a route often used with inorganic materials. However, ion damage, particularly for very thin films, is an issue when it comes to this method. An alternate method, particularly to demonstrate the practicality of coatings, is to use SEM images before and after film deposition coupled with a relevant change in a device’s property. For example, Servi et al. used iCVD to deposit thin films (≈10 nm) of hydrophobic polymers on nylon membranes to be used in membrane distillation [2]. Conformal film coverage of the membrane microstructure is essential to prevent the wetting of liquid water, a critical property for this application. As seen in Figure 7a and Figure 7b, the overall structure of a nylon membrane before and after coating by iCVD shows little to no change. However, coated membranes can withstand water pressures upwards of 100 kPa before liquid water leakage whereas uncoated membranes are immediately soaked upon contact with water (0 kPa). In this work, the combination of SEM imaging with final device properties prove the conformality of the polymer films. Many applications, particularly those involving surface property changes, require retention of the precursor functionality down the depth of feature as well. Gupta et al. used iCVD to coat 10–150 nm thick perfluorodecyl acrylate films to modify the wetting properties of capillary pore membranes, as seen in Figure 7c [4]. To determine the coating conformality, electron microprobe analysis (EMPA) was used to measure the fluorine signal down the pore wall of a coated membrane and presented in Figure 7d. While the fluorine signal is detected at the bottom of the pore, the functional side wall coverage, estimated to be between 0.5 and 0.6, indicates a degree of conformality.

**Figure 8:**
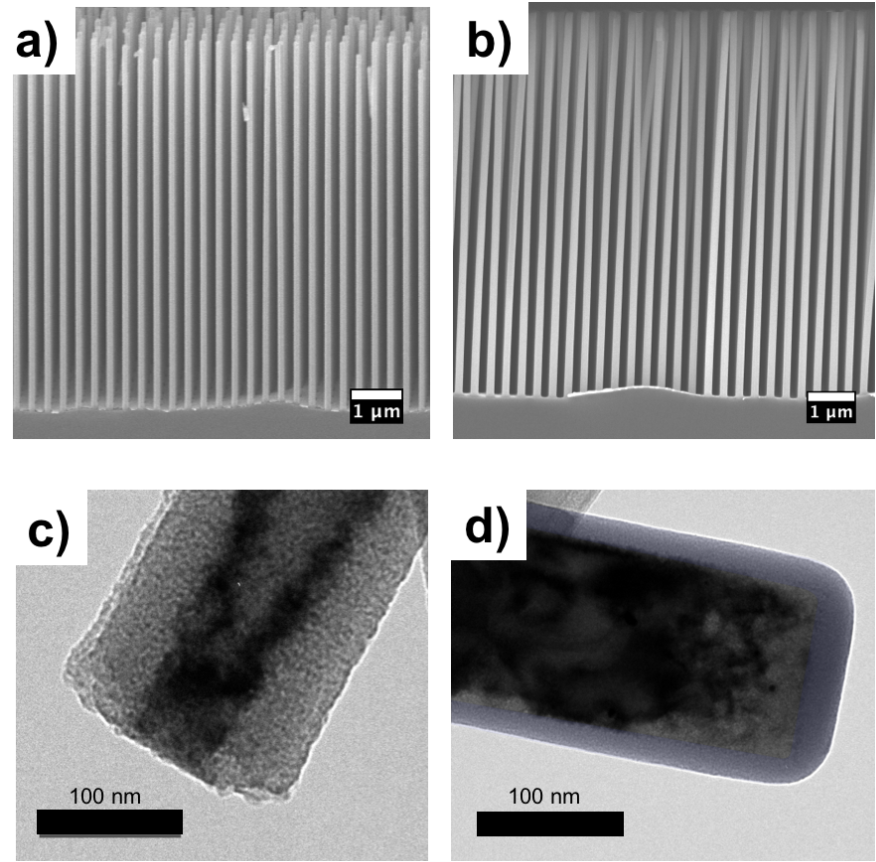
SEM images of a) uncoated ordered Si nanowire array and b) 25 nm iCVD pV4D4 coated ordered Si nanowire array. TEM image of single Si nanowire c) uncoated d) with 25 nm iCVD pV4D4 highlighted in purple.

In some cases, TEM images are necessary to verify film conformality. For instance, a conformal polysiloxane coating on an Si nanowire array is difficult to image using SEM, as creating nanowire cross sections by physical cleavage is nearly impossible. Using EMPA to determine the signal of constituent atoms fails as both the nanowire and polymer film contain nearly the same elements (Si and O). Previously unpublished work by Gleason and coworkers used a combination of SEM and TEM to verify the conformality of iCVD poly(1,3,5,7-tetramethyl-1,3,5,7-tetravinylcyclotetrasiloxane) (pV4D4) films on vertically aligned Si nanowire arrays. Figure 8a and Figure 8b show SEM images (Zeiss Merlin HR SEM) of the nanowire array before and after deposition, with no apparent change in wire structure except for e-beam induced electrostatic attraction between the coated wire tops. There is no thinning of the coated nanowires down the vertical axis, indicating good side wall coverage. TEM samples were made by sonicating the nanowire arrays in IPA to create a nanowire solution. A drop of solution on a TEM grid allows for wire dispersal and subsequent imaging. Figure 8c and Figure 8d show TEM images (FEI Tecnai G2 Spirit TWIN) of an uncoated and coated Si nanowire. The false colored amorphous layer is a ≈25 nm pV4D4 film exhibiting good SWC of approximately 0.75 within the imaged section. For very thin films, conformal protection requires that the deposited film has a smooth, pin-hole free morphology, with the root mean square roughness much smaller than film thickness.

### Applications

**Figure 9:**
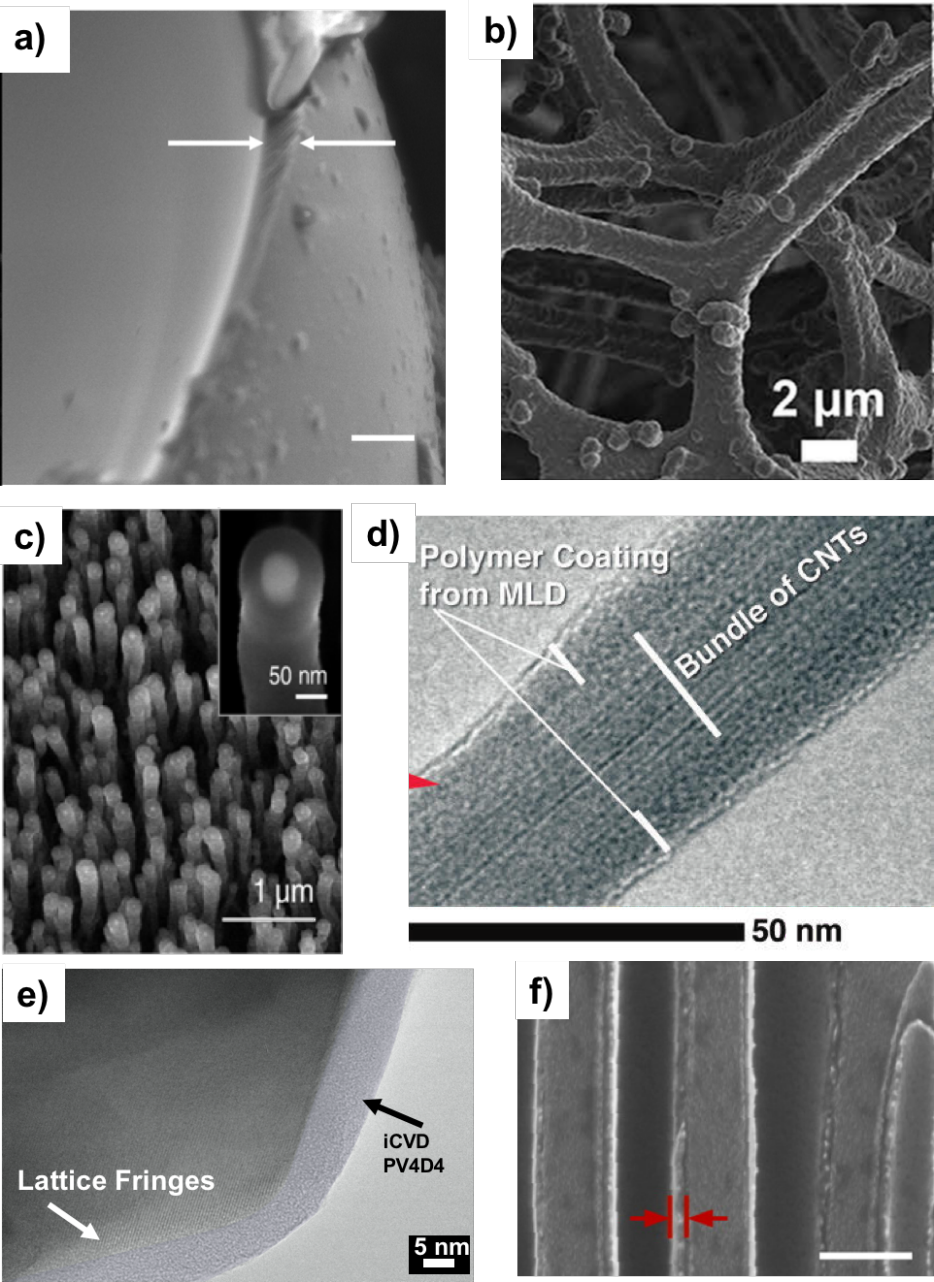
a) SEM image of silica micro-bead with conformal iCVD pHEMA coating, b) SEM image of commercial sponge with pHDFMA coating, c) SEM image of carbon nanotube forest with iCVD PTFE coating, d) TEM image of CNT bundle coated with MLD alucone coating, e) TEM image of lithium spinel oxide particle coated with iCVD pV4D4 coating, f) SEM image of NAA electrode with polythiophene coating (scale bar 200 µm). a) Reprinted with permission from [29], copyright 2008 American Chemical Society. b) Adapted from [46], Copyright 2016 Nature Publishing Group, published in [46] under a Creative Commons CC-BY license, http://creativecommons.org/licenses/by/4.0/, c) Reprinted with permission from [43], copyright 2003 American Chemical Society. d) Reprinted with permission from [40], copyright 2013 American Chemical Society. e) Reprinted with permission from [48], copyright 2016 John Wiley and Sons. f) Adapted with permission from [42], copyright 2014 American Chemical Society.

To date, a variety of conformal polymer thin films have on many substrates from nanometer length to sub millimeter length scales, as summarized in Table 1. These films have found utility in a diverse array of applications. Several biological applications have found uses for vapor deposited polymer thin films. For instance, Baxamusa et al. used iCVD to deposit conformal poly(hydroxyethyl methacrylate) hydrogels silica microspheres for biological sensors, as seen in Figure 9a [29]. Lahann and coworkers used parylene CVD to form thin films of poly[(*p*-xylene-4-methyl-2-bromoisobutyrate)-co-(*p*-xylene)] which served as a conformal initiating layer for atom transfer radical polymerization to produce conformal brushes that controlled protein adsorption [16]. Martin et al. used iCVD deposited conformal coatings of poly(dimethylaminomethylstyrene) on nylon fabric as antimicrobial agents again *E. Coli* and *B. subtilis*, as shown in Figure 6f [27]. Xu et al. demonstrated the benefit of iCVD over plasma enhanced polymer CVD both in conformality and functional group retention for the deposition of conformal sensing molecules on microfluidic devices [30]. This concept was later used to enable PDMS-free microfluidic devices for oxygen-free flow-lithography, a process that can generate multifunctional micro and nano-particles [31]. Finally, O’Shaughnessy et al. showed conformal coatings of iCVD grown poly(1,3,5-trivinyl-1,3,5-trimethylcyclotrisiloxane) (pV3D3) for biopassive insulation of neural nodes [28].

**Table 1:** Vapor deposited conformal polymer films by substrate, relevant length scale, method, and polymer chemistry.

Substrate	Width	Aspect ratio (H/W)	Method	Film chemistry and thickness	Ref

Si trench	300 nm	1.67:1	paryleneCVD	halogenated poly(*p*-xylene) 100 nm	[32]
	1 µm	5:1	iCVD	poly(methacrylate)	[8]
	500 nm	10:1	paryleneCVD	parylene-N, 200 nm	[33]
Si cantilever overhang	14 µm 1 µm opening	1:14	paryleneCVD	parylene-N, 200 nm	[33]
	20 µm 3 µm opening	3:20	iCVD	poly(tetrafluoroethylene), 300 nm	[34]
Vertical pores	3 µm	80:1	iCVD	poly(pefluorodecyl acrylate), 250 nm	[4]
	50 nm	400:1	iCVD	poly(divinyl benzene), 20 nm	[3]
Gold wires	50 µm	20:1	iCVD	poly(trivinyl-trimethyl cyclotrisiloxane), 3 µm	[28]
Nylon fibers	10 µm	100:1	iCVD	poly(dimethylaminomethyl styrene), 200 nm	[27]
PDMS micro-pillars	22 µm	2.9:1	iCVD	poly(hydroxyethyl acrylate), 1 µm	[35]
Glass microspheres	25–32 µm	1:1	iCVD	poly(glycidyl methacrylate) 135 nm	[36]
Rose petal micro-molds	20 µm	1:1 + nano-texture	iCVD	poly(glycidyl methacrylate), poly(pefluorodecyl acrylate) 500 nm	[37]
Particles	120 nm	1:1	MLD	poly(aluminum ethylene glycol) 13 nm	[38]
	200 nm	1:1	iCVD	poly(meta-diethynylbenzene) 13 nm	[19]
Bulk Ag nanowires	60 nm	166:1	iCVD	poly(tetravinyl-tetramethyl cyclotetrasiloxane), 10 nm	[39]
Bulk carbon Nanotubes	20 nm	750:1	MLD	glycercol alucone, 10 nm	[40]
Nano trenches	200 nm	2:1	oCVD	poly(3,4-ethylene dioxythiophene)	[41]
NAA	200 nm	285:1	oCVD	poly(thiophene), 30 nm	[42]
Vertically aligned Si nanowires	150 nm	50:1	iCVD	poly(tetravinyl-tetramethyl cyclotetrasiloxane), 25 nm	this work
Vertically aligned carbon nanotubes	50 nm	40:1	iCVD	poly(tetrafluoroethylene), 50 nm	[43]
	8 nm	10,000:1	oCVD	poly(3,4-ethylene dioxythiophene), 10 nm	[44]
	100 nm	20:1	iCVD	poly(methacrylic acid-co-ethylene glycol diacrylate), 50 nm	[45]

Several situations requiring the formation of composite structures have benefited from iCVD deposited polymer films. The aforementioned work by Servi et al. showed how conformal poly(divinyl benzene) coatings allowed only water vapor transport through membranes used in membrane distillation [2]. Im and coworkers fabricated self-cleaning, superamphiphobic sponges by coating poly(heptadecafluorodecyl methacrylate) on a commercial sponge using iCVD [46]. Figure 9b shows the iCVD coating conformally covering the sponge’s microstructure. Previously, Lau et al. demonstrated conformal coverage of iCVD grown fluoropolymers on vertically aligned carbon nanotube (CNT) forests to prevent capillary densification as seen in Figure 9c [43]. These coated nanotube forests were later shown to be beneficial to flexographic printing by Hart et al. [47]. Brown et al. showed that MLD could create 10 nm, conformal aluminum alkoxide derivative films on CNT sheets, as seen in Figure 9d, to create a composite material with 4 times the Young’s Modulus of a bare CNT sheet [40].

Emerging applications for ultrathin polymer films on nanostructured high aspect ratio structures include various energy storage devices and soft electronics. For instance, silicon based anodes are of interest for lithium ion batteries since Li–Si alloys have an incredibly high gravimetric lithium storage capacity. He at al. have used MLD to encapsulate Si nanoparticles with alucone for this application [49]. The alucone layer prevents the formation of a resistive secondary electrolyte interphase (SEI), thus yielding improved electrode performance. Gleason and coworkers, having previously shown pV4D4 as potential solid electrolyte, are exploring the Si nanowire assembly in Figure 8a as a route toward anodes for micro lithium ion batteries [39]. Figure 9e shows a corresponding, conformal pV4D4 coating on a lithium spinel oxide particle, a material that can be used as a cathode for micro lithium ion batteries. Composite electrodes for supercapacitors have been developed by forming pseudo-capacitive, conjugated polymer thin films on various electrodes such as vertically aligned CNTs, aligned graphene flakes, and nano-porous anodized alumina (NAA) [42,50–51]. Figure 9f shows a conformal oCVD synthesized polythiophene coating on a NAA electrode. In soft electronics, conformal dielectric iCVD films have found uses in both field effect transistors and non-volatile memory [7,52].

## Conclusion

In summary, vapor based polymerization techniques, such as parylene CVD and iCVD, yield much better conformal thin polymer films on high aspect ratio structures than traditional solution methods. Different categories of monomers are associated with the conformal polymer CVD methods discussed here. Thus, the type of resulting polymeric film desired is one criterion for selecting between the methods. The deposition rate and reactor conditions are other considerations in selecting between the methods. In all cases, depositions must operate under regimes with low reactive molecule sticking coefficients to ensure step coverage and side wall approach unity. To date, the iCVD method has shown the highest rate of vapor depositing conformal polymeric films. The degree of film conformality is typically assessed using a combination of electron microscopy and other characterization techniques. A diverse array of applications have benefited from conformal polymer films including, but not limited to, separation processes, biomedical devices, and micro/nano electronic and energy storage devices.
